# Measurement in saliva from neurotypical adults of biomarkers pertinent to autism spectrum disorders

**DOI:** 10.4155/fso.15.70

**Published:** 2015-11-01

**Authors:** Helen V Ratajczak, Robert B Sothern

**Affiliations:** 1 Edmond Enterprises, Danbury, CT 06810, USA; 2 College of Biological Sciences, University of Minnesota, St Paul, MN 55108, USA

**Keywords:** autism, biomarkers, measurement, patient-specific therapy, saliva

## Abstract

**Aim:**

Measure biomarkers pertinent to autism in saliva from humans.

**Materials & methods:**

At 7:30 PM (reading instructions) and 8:30 PM (hearing instructions), neurotypical adults (6 M, 6 F) each spat into tubes containing protease inhibitors. Cells were counted, samples aliquoted, frozen and thawed. Rationale was given for choice of biomarkers. ELISA: CD26, IL-12, carnitine, C4B, GSH, GSSG, MT-2, testosterone, IFN-γ. Mass spectrometry: cystine, glutamine, glutamic acid, GABA, serotonin. Electrochemiluminescentimmunoassay: cortisol. Radioimmunoassay: melatonin.

**Results:**

Cells averaged 2.16 × 10^6^/ml. M > F: CD-26, C4B, MT-2. Testosterone, cortisol. Glutamine, glutamic acid, IFN-γ, melatonin and GSSG were measurable. Remaining biomarkers were measured in <50% of samples. Concentrations were equal at both times.

**Conclusion:**

Saliva can be collected by literate individuals without added instruction. Ten biomarkers were measurable.

**Figure 1.**
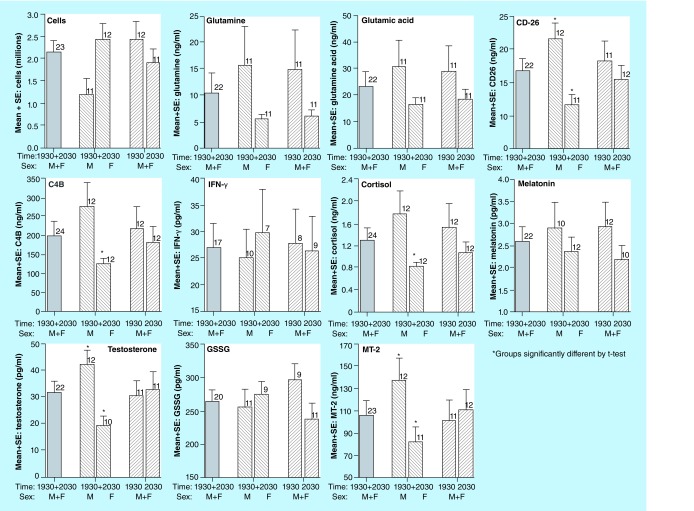
Illustration of the comparison of cell counts and concentrations of ten biomarkers that are the most likely potential candidates for use in future studies based on the detection of each analyte in at least 75% of the saliva samples collected (also see Table 5).

Oxidative stress is central to the manifestation of autism [1]. This mechanism, along with immune glutamatergic dysfunction and pineal gland malfunction, provide ‘unifying concepts’ for a contributing cause of autism spectrum disorders (ASD) [2]. More than 70 biomarkers have been shown to be present in abnormal concentrations in autistic individuals, compared with age- and sex-matched controls [2]. However, it is doubtful if one single mechanism, one single unifying concept or one single biomarker will be adequate to diagnose and/or ascertain the degree of autism. This report documents our ability to measure biomarkers pertinent to autism in human saliva, and provides a basis for an objective measure of autism.

Jepson helped define the areas of the body in which autism is manifest: ubiquitous, gastrointestinal, immunologic, neurologic and toxicologic [3]. Nevertheless, the choice of biomarkers to define autism is very difficult. The rationale for the use of each of the biomarkers is given below. The referenced studies involved children with autism compared with age- and sex-matched controls.

## Rationale of choice of biomarkers (↑ or ↓ denotes an increase or decrease found in ASD)

### Ubiquitous

#### Carnitine

↓ (serum). Concentrations of free and total carnitine were significantly reduced in a study of 145 autistic children between the ages of 2 and 13 years. Values for control individuals, age and/or gender specific, were used to create a normative range. The deficiency of carnitine, accompanied by slight elevations in lactate and significant elevations in alanine and ammonia, is a sign of mitochondrial dysfunction [4–10]. Recent studies suggest that dysregulation of carnitine metabolism may be important in nondysmorphic autism [5]. This study included 2904 individuals with simplex autism, 909 male probands from male–male sibling pairs, and over 8000 controls. Confirming carnitine's involvement with autism is the double-blind, placebo-controlled studies that used carnitine along with other treatments of mitochondrial disease, resulting in improved core and associated autistic symptoms [6]. Reduced levels of serum carnitine in autistic children have been shown to be related to gastrointestinal manifestations [7]. The autistic group included 78 males and 22 females aged between 3 and 10 years. Controls, apparently healthy, were also 78 males and 22 females, aged 3–10 years, unrelated to the autistic individuals. Carnitine is an essential co-factor in the utilization of fat reserves from body stores during fasting and stress. It plays a key role in the transport of long-chain fatty acids into the mitochondria where they undergo β-oxidation in energy production.

#### Glutamine, glutamic acid & gamma amino butyric acid

↓ (platelets, blood). Autoradiography showed an abnormality in the glutamine, glutamic acid & gamma amino butyric acid (GABA) system in autism [8]. An imbalance in the GABA system could contribute to behavioral deficits in autism. Giving benzodiazepines, which are GABA receptor agonists, to some individuals with autism results in an adverse aggressive response [9]. The serotonergic system is intimately interconnected with GABAergic and glutamatergic neurons throughout the brain. Serotonin, a precursor of melatonin, is converted to N-acetylserotonin in the pineal gland by the rate-limiting enzyme arylalkylamine N-acetyltransferase (AA-NAT), followed by the conversion of N-acetylserotonin to melatonin by acetylserotonin methyltransferase. There is a relationship among glutamine, GABA and social cognition in autism [10].

### Gastrointestinal system

#### Dipeptidyl peptidase (DPP IV)/CD26

↓ or absent in autistic individuals (urine). DPP IV is the only known enzyme to break down casomorphine. This marker is known as CD26 on T-lymphocytes. The absence of CD26 could explain the opioid-like casomorphine found to be elevated in urine from autistic patients [11]. There are alterations in plasma DPP IV in autistic individuals [12]. In addition, there is influence of candidate polymorphisms on the DPP IV and μ-opioid receptor genes expression in the aspect of the β-casomorphin-7 modulation functions in autism [13]. There is controversy about urinary opioid peptides, with MALDI-TOF confirmation of absence of these peptides in children with autism. However, HPLC showed peaks in locations at which opioid peptides might be expected to be found [14]. The main idea of the upload-excess theory suggests that excessive levels of incompletely metabolized peptides from foods that contain proteins, gluten and casein pass through the intestinal and blood–brain barriers into the brain, where they directly regulate transmission in all the main neurotransmission systems or form ligands for peptidase enzymes that would normally hydrolyze naturally-occurring opioid peptides [15].

### Immunologic system

#### Complement component C4B

↓ (blood) [16]. The deficiency of C4B in the blood correlates with the complement *C4B* gene null allele being more frequent in individuals with autism [17]. *C4B* is necessary in the activation of the classical complement pathway that results in lysis [18]. With deficient C4B, the immune system is partially compromised. The link of C4B null allele to autism and to a family history of autoimmunity is described [19].

#### IFN-γ & Interleukin-12 (IL-12)

↑ (plasma) [20,21]. IFN-γ and IL-12 regulate the function of immunoregulatory T cells, especially CD4^+^ T helper cells. The increase of these two biomarkers may suggest a defect of immunoregulatory T cells in autism. In addition, the increase may be relevant to autoimmunity in autism because IFN-γ is a cytokine of Th-1 cells, and antigenic stimulation of Th-1 cells has been implicated in the autoimmune pathogenesis of autism. IL-12 selectively stimulates Th-1 cells and initiates pathogenesis of organ-specific autoimmune diseases. The increased midgestational IFN-γ and interleukins in women bearing a child with autism shows the influence of these biomarkers during gestation [20]. Serum IL-12 has been measured in autistic children [21]. IFN-γ is proposed as a biomarker in autism [22].

### Neurologic system

#### Cortisol

↓ (serum) [23]. Decreased cortisol in serum suggests an abnormality in the hypothalamo-pituitary-adrenal axis in individuals with autism. Cortisol has an important role in proper emotional development and functioning. Cortisol is considered to be a marker (prominent and consistent) rhythm in normal individuals, with peak production in the morning [24], but shows an aberrant circadian rhythm in autistic individuals. In addition, autistic children and their neurotypical controls both showed increased salivary cortisol in anticipation of re-exposure to a perceived stressor [25]. This circadian study compared cortisol levels at different times of day. Evening values for children with autism showed a gradual decrease over the course of sampling in the morning and were consistently elevated in comparison with the neurotypical group in the evening. Autistic individuals show clear dysregulation of the circadian rhythm in autism. Autistic children between the ages of 3 and 10 years showed increased serum cortisol response to stress compared with neurotypical controls [26]. In a study of autistic children between 7 and 12 years of age, low-functioning autistic children had higher mean salivary cortisol levels than the high-functioning autistic group, which did not differ from neurotypical control children [27]. Adolescence is a period of great physiological, psychological and social change. During adolescence, there are heightened diurnal basal cortisol levels in addition to higher cortisol in response to perceived stressors. It is a time of increased vulnerability in people already prone to enhanced physiological arousal and poor adaption to change, such as autism. Adolescents with autism show more variable diurnal regulation and increased response to stress [28].

#### Melatonin

↓ (plasma) [29]. Melatonin is produced at night in the dark by the pineal gland and is a key regulator of circadian and seasonal rhythms. The acetylserotonin methyltransferase gene, encoding the last enzyme of melatonin synthesis, is deleted in some individuals with ASD. Melatonin has a crucial role in human cognition and behavior. In autistic individuals, melatonin has a dampened circadian rhythm [29]. In addition, the amount of melatonin is decreased in individuals with autism [30]. There is a feed-sidewards interaction among the various hormone-producing glands [31]. When one hormone is out of synchronization, all the other hormones are affected. Melatonin has been shown to be beneficial in treatment of children with developmental disabilities [32]. In addition, others describe melatonin as a treatment for sleep problems in children with autism [33].

#### Serotonin

↑ (platelets, brain, blood, urine) **↓** (brain, cerebral spinal fluid). Platelet hyperserotonemia is generally considered the most robust and well-replicated biological finding in autism [34]. The relative concentration of serotonin may be high or low in autistic individuals compared with controls, but different in different parts of the brain in addition to different parts of the body. The differences in serotonin levels in autistic individuals have been documented [35,36]. It is important to note that in some individuals with autism, serotonin is low in the cerebral spinal fluid but elevated in blood and platelets. In addition, pregnancy is a critical time in the life of a human, with accompanying symptoms such as hypertension, gestational diabetes and depression having significant consequences in the fetus. During such symptoms, cortisol is increased in the mother, and plays an important role in causing dysregulation of serotonergic signaling through elevating the serotonin reuptake transporter production in the developing brain [37]. Cortisol, serotonin and testosterone are key regulators of social aggression [38]. Serotonin is a precursor of melatonin.

#### Testosterone

↑ (serum) ↑ (prenatal) A study involving 92 male and female adult subjects, confirmed a positive correlation between salivary testosterone and autism [39]. The effect of testosterone on autism begins in the fetus. Studies of amniotic testosterone in humans suggest that fetal testosterone is related to specific sexually dimorphic aspects of cognition and behavior. It is suggested that autism may be an extreme manifestation of some male-typical traits, especially cognition and neuroanatomy. Influence of elevated fetal steroidogenic activity is associated with autism [40]. Concentrations of progesterone, 17a-hydroxy-progesterone, androstenedione and testosterone (sex steroids) and cortisol were positively associated with each other and were elevated in 128 amniotic fluid samples of males born between 1993 and 1999 who later were diagnosed with autism, Asperger syndrome or pervasive developmental disorder not otherwise specified compared with typically developing controls. This is the first direct evidence of fetal steroidogenic activity in autism. Studies of amniotic testosterone in humans suggest that fetal testosterone is related to specific (but not all) sexually dimorphic aspects of cognition and behavior [41]. The extreme male brain theory of autism is proposed by Baron-Cohen, linking autism with the male trait of systemizing as opposed to the female trait of empathizing [42]. It has been suggested that this gendering of certain capacities or aptitudes in the human population is unpersuasive, and could have serious consequences for treatment and services for girls and women on the autism spectrum [43]. An extreme female brain theory of psychosis in adults with autism with or without co-morbid psychosis is described, concluding that the bias for empathizing over systemizing may be linked with the presence of psychosis in people with autism. In women with autism and psychosis the link between mania/hypomania and an empathizing bias was greater than in men with autism [44]. There is a significant negative correlation between testosterone and whole-blood serotonin in autistic individuals [45]. The transsulfuration and androgen pathways interact, stemming from the fact that a critical regulatory step in the androgen pathway involves the metabolite dehydroepiandrosterone (DHEA). DHEA is a key initial regulatory metabolite in the androgen synthesis pathway, which can be converted down the androgen pathway toward testosterone or toward the normally favored storage molecule DHEA-sulfate. Marked elevation of adrenal steroids, including DHEA, were measured in saliva of prepubertal autistic children [46].

### Toxicologic system

#### Cysteine

↓ (plasma) [47]. Cysteine, an essential amino acid, is decreased in autistic children (who also have a decrease in methionine and S-adenosylmethionine-regulated cystathionine-gamma-synthase activity), premature or newborn infants, and subjects stressed by disease. Cysteine is the rate-limiting amino acid for glutathione synthesis [48]. There is a possible cyclical interaction between the methionine cycle-transsulfuration and androgen pathways in some children with autism. Significantly increased serum total testosterone was correlated with a significant decrease in plasma cysteine, reduced glutathione, methionine, cystathionine and homocysteine [49]. Because cysteine contains the thiol group (SH), it is the most chemically reactive natural amino acid found in cells. Protein thiols represent a larger active redox pool than glutathione, and are thus likely to be directly involved in the cellular defense against oxidative stress [50]. In extracellular plasma, the cysteine/cystine (thiol/disulfide) redox couple provides the redox environment for circulating immune cells. Also, in the plasma, cysteine redox ratios were decreased in autistic children [51].

#### Glutathione

↓ (plasma) [47]. Glutathione (GSH) (reduced or total) is a key component of the methionine cycle and is the predominant low-molecular-weight thiol in animal cells [48]. GSH redox ratios were decreased in autistic children [51]. GSH plays important roles in antioxidant defense, nutrient metabolism and regulation of cellular events, including gene expression, DNA and protein synthesis, cell proliferation and apoptosis, signal transduction, cytokine production, immune response and protein glutathionylation. GSH deficiency contributes to oxidative stress, which plays a key role in aging, and the pathogenesis of many diseases, including cancer, heart attack, stroke, diabetes and autism. The GSH/GSSG (thiol/disulfide) redox couple is the major mechanism for maintaining the intracellular microenvironment in a highly reduced state needed for detoxification. The cysteine/cystine redox potential has been shown to be more oxidized than the intracellular GSH/GSSG, and is regulated independently [51].

#### Oxidized glutathione (GSSG)

↑ (plasma) [47]. The consistent decrease in cysteine and GSH concentrations and GSH/GSSG in autistic children suggests an increased vulnerability to oxidative stress. GSH/GSSG is the most important redox couple and plays a crucial role in antioxidant defense, nutrient metabolism and the regulation of pathways essential for whole body homeostasis [48].

#### Metallothionein (MT-2)

↓ (blood, urine) [52]. Blood and urine from a total of 503 patients with autism, Asperger's syndrome or atypical autism were compared with those from neurotypical controls in analysis of heavy metals (it should be noted that [52] is from a meeting, and the data were not peer-reviewed). The Cu/Zn ratio was severely elevated in 85% of the patients. In addition, 99% showed evidence of a metal-metabolism disorder, suggesting a problem with metallothionein. Metallothionein is intimately involved in the development and function of the immune response, development of neurons and in detoxification of heavy metals. These functions are involved in many of the classic symptoms of autism, including gastrointestinal tract problems, an enhanced sensitivity to toxic metals and abnormal behaviors. A report of altered heavy metals and transketolase in autistic spectrum disorder documents significant change in deposition of arsenic, mercury, copper and iron in autistic children [53]. A review of the neuroprotective and neuroregenerative properties of metallothioneins is given [54].

### Hypothesis

We propose that a composite of biomarkers may be able to measure autism and define its severity. Longitudinal analysis of the array will be used to guide therapy and measure the effect of therapies. Some evidence that this approach might be successful is given by the improvement in symptoms of individuals with autism when treated with melatonin [55]. Also, James *et al*. [47] showed that giving those biomarkers to individuals with a deficit in the same biomarkers helped autistic individuals. For example, every component of the methionine cycle was shown to be in aberrant concentration in autistic subjects compared with control individuals. Plasma concentrations of methionine, S-adenosylmethionine (SAM) and homocysteine were significantly lower and S-adenosylhomocysteine (SAH) and adenosine concentrations were significantly higher than those in control children, while SAM:SAH was almost 50% lower in autistic children. James's nutritional intervention trial (oral supplementation of folinic acid and betaine) improved the concentrations of methionine, SAM, homocysteine, cystathionine, cysteine and total GSH (tGSH) and increased SAM:SAH and tGSH:GSSG. In addition, the high SAH and adenosine seen at baseline decreased. A second intervention added an injectable form of methylcobalamin (Vitamin B-12) to the above regimen. This further decreased those concentrations of adenosine and GSSG to normal levels, and increased the concentrations of those biomarkers that were too low (methionine, cysteine, tGSH, SAM:SAH and tGSH:GSSG) to normal concentrations. A list of parent ratings of behavioral effects of biomedical interventions in autistic individuals has been recorded [55]. Examples include 54% of 2738 autistic individuals improved when treated with zinc, 66% of 1687 improved when treated with melatonin and 72% of 899 improved when treated with methyl B12. These studies recorded effects of treatment of autistic individuals. Control individuals were not treated.

### Current study

In order to probe into those biomarkers identified with autism, we studied at least one biomarker from each of the systems of the body in which autism is manifest: gastrointestinal, immunologic, neurologic, toxicologic and ubiquitous (e.g., those biomarkers involved in the entire body) [3]. These biomarkers are also involved in unifying concepts of autism: increased vulnerability to oxidative stress, immune glutamatergic dysfunction and pineal gland malfunction [2].

The body fluid chosen for the measurement of biomarkers was saliva. Biomarkers have been identified in saliva from autistic individuals [56–58]. Collecting either blood or urine is more stressful to the subject than collection of saliva [58]. Saliva can be used as an adjunctive diagnostic aide for measurement of oxidative stress in autism. Also, salivary glutathione is a powerful predictor of autism spectrum disorder [59]. One of the biomarkers shown to be lower in concentration in individuals with autism is cortisol [23]. It should be noted that stress causes the amount of cortisol to be produced at a greater concentration [60]. This phenomenon is also present in autistic children, demonstrated by production of increased cortisol in anticipation of re-exposure to a perceived stressor [25,61]. As previously mentioned, it is known that alteration in production of one hormone affects the production of other hormones, caused by a feed-sidewards interaction [31]. Therefore, when choosing a body fluid for measurement of biomarkers, it is important to avoid stress as much as possible.

Our initial research used saliva from neurotypical adults to measure 16 biomarkers pertinent to autism. Assays included ELISA and MS. Because these methods were not sufficiently sensitive for some of the biomarkers, other more sensitive methods were utilized (see ‘Materials & methods’ section).

### Authorization

This study was authorized by the New England Independent Review Board, Newton, MA, USA, and was performed under the guidance of Ms Ann McLellan, Quality Assurance Officer, and in accordance with the ethical standards laid down in the 1964 Declaration of Helsinki and its later amendments regarding ethical principles for medical research involving human subjects. All persons gave their informed consent in writing prior to their inclusion in the study.

## Materials & methods

### Subjects

Twelve subjects (six men and six women) were screened prior to admission to the study, and all met the inclusion/exclusion criteria: between 21 and 80 years of age and free of neurological disorder or pathogenic disease, with no oral problems that would interfere with salivary sampling. No female was pregnant or lactating. Males included three Caucasians, one African–American and two Asians; females included four Caucasians and two Asians.

### Sample collection

The twelve neurotypical healthy adults gave samples of saliva at two separate times (7:30 and 8:30 PM, or 19:30 h and 20:30 h, military time) in order to ascertain if production of biomarkers would be similar after subjects read instructions alone compared with having been given instruction by the principal investigator. In addition, at these times the concentration of two of the chosen markers for which the circadian rhythm has been established, cortisol and melatonin, is usually lowest within a period of 24 h in blood, urine and saliva [62,63]. If these biomarkers can be measured, it is an indication that greater levels produced at different times within the 24-h day will also be able to be measured. Also, similar measurements an hour apart provide proof that approximately equal concentrations are present within that span of time, allowing flexibility in timing of collection of specimens for future chronobiological studies.

Each subject documented that he/she refrained from eating, drinking, brushing or flossing his/her teeth for 1 h prior to the first collection at 19:30 h. Then he/she washed their hands with soap (Ivory Liquid) and water, and sat down. Each drank 8 ounces of tap water from a styrofoam cup. Then he/she spit into a 50 ml polypropylene conical centrifuge tube containing 50 μL of a protease inhibitor (Cocktail, EDTA-Free, Thermo Scientific Halt Protease Inhibitor Cocktails, Catalog #78425) until at least 5 ml (indicated by a black mark on the outside of the tube) were collected. This procedure was repeated by each subject an hour later at 20:30 h for a total collection of 24 saliva samples. Each sample of saliva was gently mixed, and the cells in each sample were counted prior to making 10 aliquots of 0.5 ml each. All tubes used were polypropylene. Aliquots and the original tube were then frozen at -80˚C.

### Laboratory analyses

#### Cell counts

Cells were counted with the aid of a Cellometer Cell Counter (Nexcelom Bioscience).

#### Biomarkers

Each assay utilized an aliquot of each sample the first time it was thawed. Sixteen biomarkers were analyzed: glutamine, glutamic acid, CD-26, C4B, IFN-γ, MT-2, testosterone, IL-12, Carnitine, GSH, GSSG, cystine, GABA, serotonin, cortisol and melatonin.

ELISAs, conducted in triplicate (total of 36 replicates at each time point), were used to measure the biomarkers listed in Table 1. Only one ELISA, that for CD26, was designed for measuring quantities of the biomarker in saliva. The other kits were designed for measuring the biomarkers in plasma, serum or urine. Because concentrations of biomarkers are less in saliva, in general, than in serum or plasma [62,63], the standard curve was extended for four additional serial dilutions to enable measurement of low concentrations. The manuals accompanying the ELISA kits gave information for the minimum detectable dose (MDD, the lowest dose that could be distinguished from zero) and the lowest limit of quantitation (LLOQ, the lowest point in the standard curve).

In Table 2, the MDD is that from the manual accompanying each ELISA kit, while the LLOQ is the lower limit of quantitation determined by extension of the standard curve by four serial dilutions.

#### Mass spectrometry (Shimadzu LC System)

##### Amino acids

The aTRAQ™ Reagent Kit 50 Assay (AB Sciex Catalog # 4442668) was used to measure cystine, glutamine, glutamic acid and GABA (μM/ml). These assays were run in duplicate, with a total of 24 replicates at each time point.

##### Hormones

Serotonin, cortisol, melatonin and testosterone were measured from one sample of each specimen, with a total of 12 measurements at each time point. Analytes used are listed in Table 3.

#### More sensitive methods

Because the original methods were not sensitive enough to measure some of the biomarkers in the majority of the samples, we had another laboratory use more sensitive methods.

NeuroScience, aka The Pharmasan Lab Inc. (WI, USA), measured three of the hormones by more sensitive methods: cortisol (Electrochemiluminescent Immuno Assay), melatonin (Radio Immuno Assay) and testosterone (Enzyme-linked Immunosorbent Assay) (see Table 4).

##### Glutathione forms

Glutathione (GSH) rapidly oxidizes to GSSG and detection by ELISA was unsatisfactory.

## Results

There were 12 samples at 19:30 and 12 samples at 20:30 collected for analysis of cells and the 16 biomarkers. Overall averages using timepoint mean values for each biomarker from males and females (M+F) at both collection times (19:30 + 20:30), and averages from M+F at 19:30 versus 20:30 and M versus F at 19:30 + 20:30 are summarized in Table 5. Note: if triplicate values were available, means were only calculated if two or more were valid numbers (i.e., >0), but when duplicates were available, if one sample was zero, the second sample was used as a valid number. p-values from unpaired, 2-sided t-tests are listed, with any p ≤ 0.05 shown in bold.

### Cell numbers

The mean ± SE number of cells (× 10^6^/ml) for M+F overall from 23 samples was 2.16 ± 0.26 (individual range: 0.28 to 4.40). There were no significant (n.s.) differences (Table 5).

### Biomarkers

#### Ubiquitous system

Carnitine (nmol/ml) was measurable above the LLOQ in 11/23 (48%) of the total samples (5 at 19:30 and 6 at 20:30, 8 for M, 3 for F). The overall mean was 28.5 ± 18.1 (individual range: 0.60 to 201.0), 53.9 ± 38.5 at 19:30 versus 7.4 ± 3.1 at 20:30 (n.s.) and 37.7 ± 24.4 for M versus 4.0 ± 2.2 for F (n.s.).

Glutamine (μM/ml) was measurable above the LLOQ in 22/23 (96%) of the total samples (11 at 19:30 and 11 at 20:30, 11 for M, 11 for F). The overall mean was 10.4 ± 3.8 (individual range: 1.5 to 89.2), 14.7 ± 7.5 at 19:30 versus 6.1 ± 1.2 at 20:30 (n.s.) and 15.4 ± 7.5 for M versus 5.4 ± 1.0 for F (n.s.).

Glutamic acid (μM/ml) was measurable above the LLOQ in 22/23 (96%) of the total samples (11 at 19:30 and 11 at 20:30, 11 for M, 11 for F). The overall mean was 23.3 ± 5.4 (individual range: 4.2–124.1), 28.4 ± 10.1 at 19:30 versus 18.2 ± 3.7 at 20:30 (n.s.) and 30.5 ± 10.2 for M versus 16.1 ± 2.7 for F (n.s.).

GABA (μM/ml) was measurable above the LLOQ in 11/23 (48%) of the total samples (5 at 19:30 and 6 at 20:30, 8 for M, 3 for F). The overall mean was 2.40 ± 1.05 (individual range: 0.39 to 12.53), 4.19 ± 2.11 at 19:30 versus 0.91 ± 0.25 at 20:30 (n.s.) and 2.80 ± 1.42 for M versus 1.35 ± 0.70 for F (n.s.).

#### Gastrointestinal system

CD26 (ng/ml) was measurable above the LLOQ in 23/23 (100%) of the total samples (11 at 19:30 and 12 at 20:30, 12 for M, 11 for F). The overall mean was 16.7 ± 1.8 (individual range: 3.7 to 42.7), 18.1 ± 3.1 at 19:30 versus 15.4 ± 2.1 at 20:30 (n.s.) and 21.4 ± 2.5 for M versus 11.5 ± 1.6 for F (p = 0.004).

#### Immunologic system

C4B (ng/ml) was measurable above the LLOQ in 24/24 (100%) of the total samples (12 at 19:30 and 12 at 20:30, 12 for M, 12 for F). The overall mean was 199 ± 36 (individual range: 42 to 601), 216 ± 59 at 19:30 versus 181 ± 43 at 20:30 (*n.s.*) and 274 ± 64 for M versus 124 ± 18 for F (p = 0.033).

IFN-γ (pg/ml) was measurable above the LLOQ in 17/23 (74%) of the total samples (8 at 19:30 and 9 at 20:30, 10 for M, 7 for F). The overall mean was 27.0 ± 4.5 (individual range: 2.1 to 65.0), 27.8 ± 6.5 at 19:30 versus 26.3 ± 6.6 at 20:30 (n.s.) and 25.1 ± 5.4 for M versus 29.7 ± 8.2 for F (n.s.).

IL-12 (pg/ml) was measurable above the LLOQ in 3/23 (13%) of the total samples (2 at 19:30 and 1 at 20:30, 1 for M, 2 for F). The overall mean was 0.53 ± 0.16 (individual range: 0.23–0.80), 0.68 ± 0.12 at 19:30 versus 0.23 at 20:30 and 0.80 for M versus 0.40 ± 0.17 for F. T-tests for differences between sexes or timepoints could not be computed.

#### Neurologic system

Cortisol (ng/ml) was measurable above the LLOQ in 24/24 (100%) of the total samples (12 at 19:30 and 12 at 20:30, 12 for M, 12 for F). The overall mean was 1.29 ± 0.23 (individual range: 0.20–5.80), 1.53 ± 0.43 at 19:30 versus 1.06 ± 0.19 at 20:30 (n.s.) and 1.77 ± 0.42 for M versus 0.82 ± 0.08 for F (p = 0.038).

Melatonin (pg/ml) was measurable above the LLOQ in 22/24 (92%) of the total samples (12 at 19:30 and 10 at 20:30, 10 for M, 12 for F). The overall mean was 2.59 ± 0.33 (individual range: 1.00–6.90), 2.93 ± 0.54 at 19:30 versus 2.18 ± 0.31 at 20:30 (n.s.) and 2.87 ± 0.61 for M versus 2.36 ± 0.33 for F (*n.s.*).

Serotonin (ng/ml) was measurable above the LLOQ in 2/23 (9%) of the total samples (1 at 19:30 and 1 at 20:30, 0 for M, 2 for F). The overall mean was 3.63 ± 0.39 (individual range: 3.24–4.02) and 3.24 at 19:30 versus 4.02 at 20:30. T-tests for differences between sexes could not be computed.

Testosterone (pg/ml) was measurable above the LLOQ in 22/23 (96%) of the total samples (11 at 19:30 and 11 at 20:30, 12 for M, 10 for F). The overall mean was 31.7 ± 4.3 (individual range: 8.8–76.5), 30.5 ± 5.8 at 19:30 versus 32.9 ± 6.5 at 20:30 (n.s.) and 42.0 ± 5.7 for M versus 19.4 ± 3.8 for F (p = 0.005).

#### Toxicologic system

Cystine (μM/ml) was below the LLOQ in all samples.

rGSH (μg/ml) was measurable above the LLOQ in 2/23 (9%) of the total samples (2 at 19:30 and 0 at 20:30, 1 for M, 1 for F). The overall mean was 2.92 ± 2.12 (individual range: 0.80 for M vs 5.04 for F). T-tests for differences between sexes could not be computed.

GSSG (pg/ml) was measurable above the LLOQ in 20/23 (87%) of the total samples (9 at 19:30 and 11 at 20:30, 11 for M, 9 for F). The overall mean was 263 ± 18 (individual range: 42 to 601), 295 ± 24 at 19:30 versus 236 ± 24 at 20:30 (n.s.) and 254 ± 29 for M versus 273 ± 21 for F (n.s.).

MT-2 (ng/ml) was measurable above the LLOQ in 23/23 (100%) of the total samples (11 at 19:30 and 12 at 20:30, 12 for M, 11 for F). The overall mean was 106 ± 13 (individual range: 26 to 201), 101 ± 19 at 19:30 versus 110 ± 19 at 20:30 (n.s.) and 137 ± 17 for M versus 71 ± 14 for F (p = 0.009).

See Figure 1 for comparison of times of measurement and comparison of values for males versus females (M vs F) for those biomarkers that could be measured in the majority of samples. Note that Figure 1 shows values from the ELISA assay for GSSG.

Comparisons are shown between sexes and between sampling times, with statistical differences if p < 0.05 shown with an asterisk. Significant differences between sexes were found for CD26, C4B, cortisol, testosterone and MT-2, and for each of these biomarkers, the males had significantly higher levels than females. In contrast, there were no significant differences between measurement times for all M+Fs combined.

See Table 6 for comparisons of current results with those of neurotypical adults published in the literature. When converted to the same units, our data show the saliva to contain less than the literature's reports of the same biomarkers in plasma or serum, with the exception of four biomarkers (GABA, testosterone, GSH, MT-2), which were more prevalent in saliva. Although our data are limited to samples from 12 individuals tested on two occasions, the data show that some biomarkers are greater in saliva than blood, and support saliva as the body fluid of choice for measuring biomarkers pertinent to autism.

## Discussion

When designing this study, a major consideration was to keep the saliva collection process as simple as possible in anticipation of collecting from autistic subjects in a future study. Although there are many collection devices on the market, the most noninvasive and stress-free method is simply to have the subject spit into a large tube. In addition, the tubes used for collection and storage of the saliva are made of polypropylene in an effort to avoid any adherence of the saliva components to the storage vessel, as well as any possibility of breakage (if glass were used).

In an effort to eliminate biases, the current study included adults of similar ages (M 25–72 years, F 24–70 years) with only one of each sex in their 70s. Also, ethnicity was very similar (M – 3 Caucasians, 1 African, 2 Asians; F – 4 Caucasians, 2 Asians). The study is too small to determine any effect of age, ethnicity or sex.

The data produce a proof of concept that biomarkers pertinent to autism can be measured in human saliva. Further studies will need to be performed in children owing to the difficulties with saliva collection.

There were no significant differences between the two times of measurement for any analyte, albeit only 1 h apart, for both sexes combined. This finding illustrates that similar concentrations of the biomarkers were measured if the subjects read the instructions for saliva sample collection alone or if they later received instructions from the principal investigator. While this information allows for collection of saliva samples without the presence of the principal investigator, someone able to read and interpret the instructions nevertheless would need to be present, since many autistic individuals would need the presence of a literate individual to instruct and assist them in the proper manner of sample collection and handling.

While there were no differential counts of the types of cells present, an overall cell count was made, with numbers ranging from 0.28 to 4.40 × 10^6^/ml, which is consistent with the literature [77]. There was no attempt to preserve the cells, but a protease inhibitor was used to preserve the biomarkers from degradation. The saliva was frozen and stored until it was thawed for analysis. The freeze-thaw caused the cells to lyse and the samples were centrifuged prior to analysis to avoid any debris from interfering with the assay. At this point, there was no attempt to correlate the number of cells per sample with the concentrations of the biomarkers.

The saliva and the cells lining the inside of the cheeks are part of the mucosa-associated lymphoepithelial tissue. The main function of the mucosa-associated lymphoepithelial tissue is generation and dissemination of stimulated B cells (which differentiate into plasma cells and make antibodies), which subsequently require some secondary stimuli for terminal differentiation in secretory tissues. The tonsils differ from the other lymphoepithelial structures by showing considerable *in situ* differentiation to plasma cells. The cells in saliva include antigen-presenting cells (macrophages, dendritic cells and certain epithelial cells), and T- and B-lymphocytes. The T-lymphocytes help the B-lymphocytes transform into plasma cells due to environmental factors, which are regulatory signals. Other regulatory signals include lymphokines, such as IFN-γ, which assist in the making of secretory antibody. Thus, the saliva and cells present are intimately involved in the body's defense mechanisms, reacting to all things that enter the mouth [78].

It was expected that the concentration of biomarkers in saliva would be less than that in blood or urine, since the saliva is considered a filtrate of the blood [62,63]. The extension of the standard curve in ELISAs allowed measurement of smaller concentrations. Despite this, for those eight biomarkers measured by ELISA, only five could be measured in the majority of the saliva samples: CD26, C4B, IFN-γ, GSSG and MT-2. Because GSH is very unstable and oxidizes very readily, we will not measure GSH or GSSG in our future study. Similarly, mass spectrometry was not sensitive enough to measure the hormones or two of the amino acids in saliva. The Pharmasan Laboratory measured the hormones by more sensitive means: cortisol (Electrochemiluminescent Immuno Assay), melatonin (Radio Immuno Assay) and testosterone (Enzyme-Linked Immunosorbent Assay).

We measured ten biomarkers in ≥74% of the saliva samples: glutamine, glutamic acid (ubiquitous), CD26 (gastrointestinal), C4B, IFN-γ (immunologic), cortisol, melatonin, testosterone (neurologic), oxidized glutathione and Metallothionein-2 (toxicologic). Our data are consistent with the literature and provide a basis for a future study in which we plan to measure the circadian rhythm of these biomarkers. The future study will aid in producing patient-specific therapy to bring the biomarkers into synchronization and to provide homeostasis and health, lessening the symptoms of autism. This was clearly shown for melatonin [55] and the components of the methionein cycle [47].

## Conclusion

In summary, the data prove the concept that saliva is an appropriate body fluid in which to measure biomarkers important to autism. We found measurable levels in 74–100% of saliva samples from 12 healthy neurotypical adults on two occasions for 10 biomarkers important to autism: glutamine, glutamic acid, CD26, C4B, IFN-γ, cortisol, melatonin, testosterone, MT-2 and GSSG, but in <50% of samples for carnitine, GABA, IL-12, serotonin, cystine and rGSH.

## Future perspective

We plan to investigate the biorhythms of the ten biomarkers in saliva for times of lowest and highest values and extent of change over 24 h in neurotypical individuals so that ‘normal’ can be defined. Then the difference from normal exhibited by individuals with autism, hopefully at only one or two selected timepoints, can be used to determine a rudimentary objective measure of autism. For example, the circadian rhythms of melatonin and cortisol have been shown to be dampened in autism [25,29]. The data from the current study prove we can measure at least one biomarker from each system of the body in which autism is manifest. The planned biorhythm study should produce data, which, with the aid of discriminate and multiple regression analyses, will provide a rudimentary objective measure of autism. In biorhythm studies, measurements will be taken from saliva samples collected every 4 h for a total of six collections per subject in 24 h. Concentrations of biomarkers from neurotypical individuals will be used for comparison with those from subjects with autism. The premise is that measuring the amount of difference from normal will allow the possibility of ranking the severity of autism. These data will also be correlated with existing diagnoses of autism. It is hoped that this measure will provide a patient-specific guide for therapy. After more studies, the data might also be used to help diagnose the severity of autism.

**Table 1.** **Human ELISA kits used.**

**Variable (Abbreviation)**	**Kit**	**Catalog no.**	**Manufacturer**
Dipeptidyl peptidase IV/CD26	Quantikine	DC260	1
IL-12	Quantikine	D1200	1
Total carnitine	Cusabio Biotech Co., Ltd.	CSB-E13242h	2
C4B	USCN Life Science Inc.	E91305Hu	2
GSH	Cusabio Biotech Co., Ltd.	CSB-E09495h	2
MT-2	Cusabio Biotech Co., Ltd.	CSB-E13535h	2
IFN-γ	Quantikine	DIF50	1
GSSG	Cusabio Biotech Co., Ltd.	CSB-E13735h	2

^†^1: R&D Systems, Minneapolis MN 55413, USA; 2: Wuhan, Huabei Province 430223, P.R. China.

**Table 2.** **Comparisons of values detected above lowest possible levels of measurement by ELISA.**

**Biomarker**	**Units**	**n values**	**MDD**	**Values >MDD**		**LLOQ**	**Values >LLOQ**	
				**n**	**%**		**n**	**%**
C4B	ng/ml	24	1.90	24	100	0.50	24	100
CD26	ng/ml	23	0.016	23	100	0.018	23	100
MT-2	ng/ml	23	0.80	23	100	0.19	23	100
GSSG	pg/ml	23	9.38	20	87	0.29	20	87
IFN-γ	pg/ml	22	8.00	13	59	0.90	17	77
Carnitine	nmol/ml	23	0.78	8	35	0.10	11	48
GSH	μg/ml	23	0.20	1	4	0.049	2	9
IL-12	pg/ml	23	5.00	0	0	0.40	2	9

^LLOQ:^Lower limit of quantitation; MDD: Mean detectable dose.

**Table 3.** **Analytes for measurement of hormones by MS.**

**Analyte**	**Manufacturer**	**Catalog number**
Cortisol	Cerilliant, TX 78665, USA	C-106
Melatonin	Cerilliant, TX 78665, USA	M-095
Serotonin hydrochloride	Sigma Aldrich, MO 63178, USA	H9523
Testosterone	Cerilliant, TX 78665, USA	T-037
N-Acetyl-5-methoxytryptamine-d4	CDN Isotopes, Pointe-Claire, Quebec H9R 1H1, Canada	D-2952
Cortisol-d4	Cerilliant, TX 78665, USA	C-113
Testosterone-d3	Cerilliant, TX 78665, USA	T-046
Human saliva	BioReclamation, NY 11590, USA	HMSALIVA

**Table 4.** **Hormone analysis by more sensitive methods.**

**Hormone**	**Means of analysis**	**Reagent manufacturer**	**Limits of detection**
Cortisol	Electrochemiluminescent immuno assay	Roche, IN 46256, USA	0.36–30 ng/ml
Melatonin	Radio immuno assay	IBL, MN 55432, USA	1–300 pg/ml
Testosterone	Enzyme linked immunosorbent assay	DRG Diagnostics D-35039 Marburg, Germany	7.1–4500 pg/ml

**Table 5.** **Biomarker concentrations in saliva from 12 neurotypical adults (six males, six females) sampled at 19:30 and 20:30 h.**

**Category**	**Variable**	**Units**	**19:30 & 20:30**			**19:30**			**20:30**			**19:30 vs 20:30**	**19:30 & 20:30**			**19:30 & 20:30**			**19:30 & 20:30**
			**M & F**			**M & F**			**M & F**			**M & F**	**M**			**F**			**M vs F**
			**n (total)**	**Mean**	**±SE**	**n**	**Mean**	**±SE**	**n**	**Mean**	**±SE**	**t-test p =**	**n**	**Mean**	**±SE**	**n**	**Mean**	**±SE**	**t-test p =**
General	Cells	million	23(23)	2.16	±0.26	11	2.43	±0.44	12	1.92	±0.31	0.345	12	1.90	±0.39	11	2.45	±0.35	0.311
Ubiquitous	Carnitine	nM/ml	11(23)	28.5	±18.1	5	53.9	±38.5	6	7.4	±3.1	0.216	8	37.7	±24.4	3	4.0	±2.2	0.434
	Glutamine	μM/ml	22(23)	10.4	±3.8	11	14.7	±7.5	11	6.1	±1.2	0.270	11	15.4	±7.5	11	5.4	±1.0	0.198
	Glutamic Acid	μM/ml	22(23)	23.3	±5.4	11	28.4	±10.1	11	18.2	±3.7	0.358	11	30.5	±10.2	11	16.1	±2.7	0.185
	GABA	μM/ml	11(23)	2.40	±1.05	5	4.19	±2.11	6	0.91	±0.25	0.122	8	2.80	±1.42	3	1.35	±0.70	0.565
Gastro-intestinal	CD26	ng/ml	23(23)	16.7	±1.8	11	18.1	±3.1	12	15.4	±2.1	0.462	12	21.4	±2.5	11	11.5	±1.6	**0.004**
Immunologic	C4B	ng/ml	24(24)	198.9	±35.9	12	216.3	±59.4	12	181.5	±42.6	0.639	12	274.2	±63.6	12	123.5	±17.8	**0.033**
	IFN-g	pg/ml	17(23)	27.0	±4.5	8	27.8	±6.5	9	26.3	±6.6	0.874	10	25.1	±5.4	7	29.7	±8.2	0.629
	IL-12	pg/ml	3(23)	0.53	±0.16	2	0.68	±0.12	1	0.23	–	–	1	0.80	–	2	0.40	±0.17	–
Neurologic	Cortisol	ng/ml	24(24)	1.29	±0.23	12	1.53	±0.43	12	1.06	±0.19	0.327	12	1.77	±0.42	12	0.82	±0.08	**0.038**
	Melatonin	pg/ml	22(24)	2.59	±0.33	12	2.93	±0.54	10	2.18	±0.31	0.262	10	2.87	±0.61	12	2.36	±0.33	0.450
	Serotonin	ng/ml	2(23)	3.63	±0.39	1	3.24	–	1	4.02	–	–	0	–	–	2	3.63	±0.39	–
	Testosterone	pg/ml	22(23)	31.7	±4.3	11	30.5	±5.8	11	32.9	±6.5	0.781	12	42.0	±5.7	10	1 9.4	±3.8	**0.005**
Toxicologic	Cysteine	μM/ml	0(23)	–	–	0	–	–	0	–	–	–	0	–	–	0	–	–	–
	GSH	μg/ml	2(23)	2.92	±2.12	2	2.92	±2.12	0	–	–	–	1	0.80	–	1	5.04	–	–
	GSSG	pgl/ml	20(23)	262.7	±17.9	9	295.3	±24.3	11	236.1	±23.8	0.102	11	254.1	±28.5	9	273.2	±20.7	0.610
	MT-2	ng/ml	23(23)	105.5	±13.1	11	100.5	±19.4	12	110.0	±18.5	0.726	12	136.9	±17.3	11	71.2	±14.4	**0.009**

n(total) = n samples with measurable analyte level determined (n for total samples analyzable); dash (-) if analyte not detected or parameter could not be computed.

**Table 6.** **Concentration of biomarkers from the current study compared to the literature.**

**System & biomarker**	**Current study: Saliva**		**Literature: Plasma/serum**		**Age (y [n subjs])**	**Ref.**
	**Mean ± SD (units)**	**Age (y) [n data]**	**Mean ± SD (units)**	**Mean (converted units)**		
**Ubiquitous**						
Carnitine	28.52 ± 59.88 (nmol/ml)	22–80 (24)	51.5 ± 3.3 (μmol/L)	51,500 (nmol/ml)	24–48 (15)	[64]
Glutamine	10.38 ± 18.00 (μM/ml)	22–80 (22)	575 (μmol/L)	84.04 (μM/ml)	ND	[65]
Glutamic acid	23.29 ± 25.16 (μM/ml)	22–80 (22)	54.7 ± 2.16 (μmol/100 ml)	100.4 (μM/ml)	Adult (6)	[66]
GABA	1.18 ± 2.70 (μM/ml)	22–80 (22)	306 ± 79 (pmol/ml)	0.032 (μM/ml)	ND	[67]
**Gastrointestinal system**						
CD26	16.68 ± 8.68 (ng/ml)	22–80 (23)	910 (ng/ml)	910 (ng/ml)	38.5 (100)	[68]
**Immunologic system**						
C4B	198.9 ± 175.0 (ng/ml)	22–80 (24)	62.75^†^;124.56^‡^ (mg/L)	62,750^†^;124,560^‡^ (ng/ml)	ND (18)^†^ (42)^‡^	[69]
IFN-γ	26.99 ± 18.58 (pg/ml)	22–80 (22)	139.6 ± 7.9 (pg/ml)	139.6 (pg/ml)	ND (20)	[70]
IL-12	0.533 ± 0.285 (pg/ml)	22–80 (22)	60.7 (pg/ml)	60.7 (pg/ml)	60.8 ± 1.9 (30)	[71]
**Neurologic system**						
Cortisol	1.29 ± 1.14 (ng/ml)	22–80 (24)	250 ± 13 (nmol/L)	90.62 (ng/ml)	19–57 (10)	[72]
Melatonin	2.59 ± 1.54 (pg/ml)	22–80 (22)	0.26 ± 0.13 (nmol/L)	60.39 (pg/ml)	ND (32)	[73]
Serotonin	3.63 ± 0.39 (ng/ml)	24 (2)	0.92 ± 0.17 (μmol/L)	152.12 (ng/ml)	ND (16)	[73]
Testosterone	31.68 ± 20.00 (pg/ml)	23–80 (22)	18.8 ± 0.8 (nmol/L)	5.41 (pg/ml)	19–57 (10)	[72]
**Toxicologic system**						
Cysteine	0 (μM/ml)	NA (NA)	0.95 (mg/100 ml)	9500 (μM/ml)	19–50 (5)	[74]
GSH	3.34 ± 3.35 (μg/ml)	25,80 (2)	2.09 ± 1.14 (μM)	0.642 (μg/ml)	20–43 (59)	[75]
GSSG	262.7 ± 80.1 (pg/ml)	22–80 (20)	0.083 ± 0.090 (μM)	508,504 (pg/ml)	20–43 (59)	[75]
MT-2	105.5 ± 63.0 (ng/ml)	22–80 (23)	3.42 ± 2.30 (ng/ml)	3.42 (ng/ml)	41.3 ± 6.7 (28)	[76]

^†^One *C4B* gene (serum).

^‡^Two C4B genes (serum).

ND: No data, NA: Not applicable.

**Executive summary**

Biomarkers chosen for analysis are documented by others as being in significantly different concentrations in the blood or urine of subjects with autism compared with neurotypical controls.
Chosen biomarkers participate in unifying concepts of the causes of autism:
  Increased vulnerability to oxidative stress
  Immune glutamatergic dysfunction
  Pineal gland malfunction
Saliva was chosen as the body fluid because collection causes less stress than blood or urine. This results in fewer changes in concentration of the biomarkers.
Chosen biomarkers represent systems of the body in which autism is manifest.
Rationale for choice of biomarkers is given.
Future studies are designed to provide a subject-specific profile of biomarkers, with a ranking of severity of the difference of concentration of each biomarker from normal, which would comprise a rudimentary objective measure of autism.
This objective measure of autism could be a subject-specific guide for therapy.
After more comprehensive studies, the data might also be used to diagnose the degree or progression of autism.
